# A zero-dose synthetic baseline for the personalized analysis of [^18^F]FDG-PET: Application in Alzheimer’s disease

**DOI:** 10.3389/fnins.2022.1053783

**Published:** 2022-11-24

**Authors:** Christian Hinge, Otto Mølby Henriksen, Ulrich Lindberg, Steen Gregers Hasselbalch, Liselotte Højgaard, Ian Law, Flemming Littrup Andersen, Claes Nøhr Ladefoged

**Affiliations:** ^1^Department of Clinical Physiology, Nuclear Medicine and PET, Rigshospitalet, University of Copenhagen, Copenhagen, Denmark; ^2^Danish Dementia Research Centre, Rigshospitalet, University of Copenhagen, Copenhagen, Denmark

**Keywords:** deep learning, artificial intelligence, FDG, brain PET/MRI, baseline, Alzheimer’s disease, anomaly detection

## Abstract

**Purpose:**

Brain 2-Deoxy-2-[^18^F]fluoroglucose ([^18^F]FDG-PET) is widely used in the diagnostic workup of Alzheimer’s disease (AD). Current tools for uptake analysis rely on non-personalized templates, which poses a challenge as decreased glucose uptake could reflect neuronal dysfunction, or heterogeneous brain morphology associated with normal aging. Overcoming this, we propose a deep learning method for synthesizing a personalized [^18^F]FDG-PET baseline from the patient’s own MRI, and showcase its applicability in detecting AD pathology.

**Methods:**

We included [^18^F]FDG-PET/MRI data from 123 patients of a local cohort and 600 patients from ADNI. A supervised, adversarial model with two connected Generative Adversarial Networks (GANs) was trained on cognitive normal (CN) patients with transfer-learning to generate full synthetic baseline volumes (sbPET) (192 × 192 × 192) which reflect healthy uptake conditioned on brain anatomy. Synthetic accuracy was measured by absolute relative %-difference (Abs%), relative %-difference (RD%), and peak signal-to-noise ratio (PSNR). Lastly, we deployed the sbPET images in a fully personalized method for localizing metabolic abnormalities.

**Results:**

The model achieved a spatially uniform Abs% of 9.4%, RD% of 0.5%, and a PSNR of 26.3 for CN subjects. The sbPET images conformed to the anatomical information dictated by the MRI and proved robust in presence of atrophy. The personalized abnormality method correctly mapped the pathology of AD subjects while showing little to no anomalies for CN subjects.

**Conclusion:**

This work demonstrated the feasibility of synthesizing fully personalized, healthy-appearing [^18^F]FDG-PET images. Using these, we showcased a promising application in diagnosing AD, and theorized the potential value of sbPET images in other neuroimaging routines.

## Introduction

Alzheimer’s disease (AD) is a progressive, neurodegenerative disorder accountable for 50–75% of all dementia cases (Lane et al., 2018). Early diagnosis is crucial, and neuroimaging, primarily magnetic resonance imaging (MRI) and positron emission tomography (PET), has proven to be valuable for the detection of the disease in the early stages (Garibotto et al., 2017). For this, 2-Deoxy-2-[^18^F]fluoroglucose ([^18^F]FDG) is an accessible, accepted, and well-documented tracer that can depict the distribution of glucose uptake within the brain (Henriksen et al., 2016; Nestor et al., 2018; Guedj et al., 2022). Translating [^18^F]FDG uptake to clinically useful information is, however, a demanding task due to high data-dimensionality, intra-patient heterogeneity of brain morphology, and common AD mimicking conditions, such as cerebrovascular disease (Burgos et al., 2017; Choi et al., 2019). Consequently, operator-independent, quantitative readouts often complement traditional visual assessment, which has shown to improve classification rates and inter-rater agreement (Garibotto et al., 2017). More specifically, clinicians frequently interpret [^18^F]FDG-PET images through statistical comparisons of the patient with healthy control templates of similar demographics (Henriksen et al., 2016). This setup poses a challenge as a detected decrease in [^18^F]FDG uptake relative to the healthy controls is unspecific and may be due to a number of different conditions, including neurodegenerative diseases, vascular damage, diaschisis, or intrinsic anatomical differences between patient and controls stemming from age-related atrophy (Baron et al., 1986; Burgos et al., 2017; Choi et al., 2019). Consequently, there has been a growing interest in using personalized computational tools to extract diagnostic-relevant features from PET and MRI data (Garibotto et al., 2017; Burgos et al., 2021; Wu et al., 2022).

Recent advances in artificial intelligence (AI) with deep learning convolutional neural networks (CNN) have successfully been expanded to aid clinicians in neuroimaging routines (Ladefoged et al., 2020). For AD diagnostics, the traditional AI approach has been to predict the category of pathology in a patient directly from medical images (Wen et al., 2020). Although often achieving high accuracy on datasets, the classification paradigm is perhaps unfit for clinical use, as the reasoning behind disease predictions is difficult to interpret from CNNs (Choi et al., 2019). Furthermore, such models are tasked with predicting a finite number of certain diseases, so performance may be limited by the training cohort (Arbabshirani et al., 2017; Choi et al., 2019). For instance, a network trained on Alzheimer’s Disease Neuroimaging Initiative (ADNI) studies might not generalize to an unselected population of clinically referred patients as a cognitive decline may be attributed to other pathologies not represented in the dataset (Arbabshirani et al., 2017; Choi et al., 2019). Instead, emerging studies focus on addressing the shortcomings of ordinary statistical templates by utilizing neural networks for image-to-image translation (Choi et al., 2019; Sikka et al., 2021). More specifically, the methods synthesize personalized baselines that when compared against the patients’ own [^18^F]FDG-PET images produce interpretable abnormality maps more resilient to anatomical variation (Burgos et al., 2017; Choi et al., 2019). Investigations had variational auto encoders (VAE) synthesize healthy-appearing [^18^F]FDG-PET baselines tailored to the brain morphology of each patient, which enabled easy detection of metabolic abnormalities even in the case of rare disorders (Choi et al., 2019). Related studies using adversarial architectures reached a similar conclusion; personalized baselines in place of a statistical template increase the robustness of the analysis against morphological variability (Sikka et al., 2021). Concurrent with our work, a new method showed promising results by using cross-modality MRI-to-PET synthesis for personalized baselines (Sikka et al., 2021). However, since most such models rely on ADNI studies for training and testing, the clinical validity of the methodology is yet to be established on newer, high-resolution scanners.

Addressing this issue, the aim of this study was to develop and test the principle of a novel deep learning model for synthesizing an individual, healthy [^18^F]FDG-PET image from a patient’s own MR image. This was achieved by utilizing a locally acquired cohort of cognitive normal subjects with PET/MRI studies along with ADNI studies. To demonstrate the proof-of-concept in one probable application, the synthetic “zero-dose” images were used as personalized controls in the diagnosis of Alzheimer’s disease.

## Materials and methods

### Patients

#### Local cohort

**FIGURE 1:**
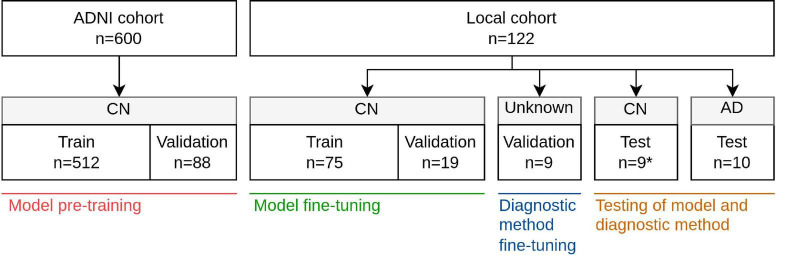
Overview of cohorts, groups, and data splits and in which of the four stages of development these were used; model pre-training, model fine-tuning, diagnostic method fine-tuning, and testing. *One subject was removed from the original 10 CN test subjects due to presence of pre symptomatic FTD3.

A total of 123 [^18^F]FDG-PET/MRI studies from 123 subjects were obtained retrospectively at Rigshospitalet, Copenhagen University, Denmark. Among these, nine subjects were of unknown diagnosis but exhibited clinically striking imaging features such as severe cortical atrophy, ventricular enlargement and/or [^18^F]FDG uptake characteristics of dementia. This “Unknown” group was used to tune the diagnostic method (Figure 1). The remaining 114 subjects had undergone evaluation at the Memory Clinic for suspected dementia. [^18^F]FDG-PET/MRI studies complemented the investigations, and in 104 of the subjects, the cognitive function was considered normal after taking educational and cultural background into account (Kaltoft et al., 2019). Although considered cognitive normal (CN), some exhibited other neurological and psychiatric disorders including, vascular infarcts, epilepsy, psychosis, and presymptomatic Huntington’s disease. Others showed significant atrophy where an underlying cause could not be determined. These 104 subjects were assembled in a CN group, which was divided into parts for model training (*n* = 75), holdout validation during training (*n* = 19), and final holdout testing (*n* = 10). Two nuclear medicine physicians examined the [^18^F]FDG-PET/MRI studies and patient journals of the test subjects, which caused the removal of a single subject with pre symptomatic frontotemporal dementia localized to chromosome 3 (FTD3). Finally, 10 subjects clinically diagnosed with dementia attributed to AD were collected in an AD group and used for testing the diagnostic application (Kaltoft et al., 2019).

The 122 subjects, 57 males and 65 females, ranged in age from 27 to 87 years with a median value of 63. All scans were performed between March 2011 and November 2019 with a fully integrated PET/MRI system (Biograph mMR, Siemens Healthineers, Erlangen, Germany), and data were extracted only in fully anonymized format in compliance to the European General Data Protection Regulation (GDPR) (Delso et al., 2011). Due to the retrospective design of the study, approval of the regional ethics committee was not required. Use of patient data was approved by the Danish Patient Safety Authority (ref. 3-3013-1513/1).

#### Alzheimer’s disease neuroimaging initiative

A part of the imaging data of this study was obtained from the Alzheimer’s Disease Neuroimaging Initiative database.^1^ The ADNI was launched in 2003 as a public-private partnership, led by Principal Investigator Michael W. Weiner, MD. The primary goal of ADNI has been to test whether serial magnetic resonance imaging, positron emission tomography, other biological markers, and clinical and neuropsychological assessment can be combined to measure the progression of mild cognitive impairment (MCI) and early Alzheimer’s disease. For up-to-date information, see http://www.adni-info.org.

[^18^F]FDG-PET images of pre-processing type CO-REGISTERED AVERAGED and T1w MR images were obtained from 340 subjects all classified as cognitive normal.^2^ Each [^18^F]FDG-PET scan was paired with the T1w MPRAGE scan closest in time, excluding pairs where the difference in acquisition date exceeded 30 days, where the subject transitioned away from CN within 5 years of acquisition date, or where no unique MRI was available for a given PET scan. Due to repeated visits, some subjects ended with multiple paired [^18^F]FDG-PET and MRI scans. The resulting cohort comprising 600 paired PET and MRI studies from 276 subjects was split, on subject level, into a pre-training segment and a validation segment, with the latter enabling early stopping (Figure 1). ADNI subject and image ID’s are listed in Supplementary Table 1.

### Imaging protocol

#### Local cohort

T1-weighted (T1w) MPRAGE images were acquired with acquisition parameters listed in Supplementary Table 2. Patients were placed head first-supine (HFS) in the scanner, and data were acquired for 10 min with reconstruction parameters: matrix size; 344 × 344 × 127 and voxel size; 0.8 mm × 0.8 mm × 2 mm. The scans had a mean post-injection time of 49 min, [interquartile range (IQR): 44 min, 52 min] and patients were administered, on average, an activity of 200 MBq [^18^F]FDG, (IQR: 198, 201). Postprocessing included reconstruction with 3D Ordinary Poisson-Ordered Subset Expectation Maximization (OP-OSEM) using four iterations, 21 subsets, and 3 mm Gaussian post-filtering. The European GDPR was fulfilled by transforming the dataset to anonymized data. [^18^F]FDG-PET images were attenuation corrected using a co-registered same-day CT (Andersen et al., 2014).

#### Alzheimer’s disease neuroimaging initiative

T1w MPRAGE and [^18^F]FDG-PET images from ADNI were acquired with various imaging systems, acquisition parameters, and reconstruction methods (see text foonote 1).

### Pre-processing

An ordinary registration and normalization scheme standardized all scans to a common space (Wen et al., 2020). The MR images were bias-corrected *via* the N4ITK algorithm, skull-stripped through HD-BET, and registered to MNI space (MNI152 NLIN 2009a symmetric) *via* ANTs Affine transformation (Fonov et al., 2009; Tustison et al., 2010; Avants et al., 2011). FSL FAST was used to segment gray matter, white matter, and cerebrospinal fluid (CSF) probability maps, and Hammersmith maximum probability regions (Hammers_mith-n30r83) were nonlinearly registered to each MRI with NiftyReg F3D (Hammers et al., 2003; Gousias et al., 2008; Fonov et al., 2009; Modat et al., 2010; Jenkinson et al., 2012). The images in MNI space, now of dimension 197 × 233 × 189, were cropped and padded to obtain a 192 × 192 × 192 isometric 1 mm^3^ space. The brain-masks were dilated by 10 mm, which was theorized to aid model predictions near the skull. Finally, the MRI signal intensity was *z*-normalized with the mean and standard deviation calculated only from voxels depicting gray or white matter. This helped ensure robustness against varying degrees of atrophy. To distinguish unmasked voxels from tissue-voxels, voxels outside the 10 mm-dilated brain-mask were assigned a value of −7.

The [^18^F]FDG-PET images were rigidly (affinely for the ADNI-cohort) registered *via* ANTs to the MR images and propagated to MNI space using the affine transformation matrices derived from the MRI pre-processing (Avants et al., 2011). The PET images were skull-stripped using the affinely propagated brain-masks and cropped and padded to the same isometric space as the MRIs. To ensure a common intensity space, each PET image was normalized by the average of the top 2% gray and white matter voxels following a 3 mm gaussian blurring. See Supplementary Table 3 for software versions.

### Model

**FIGURE 2:**
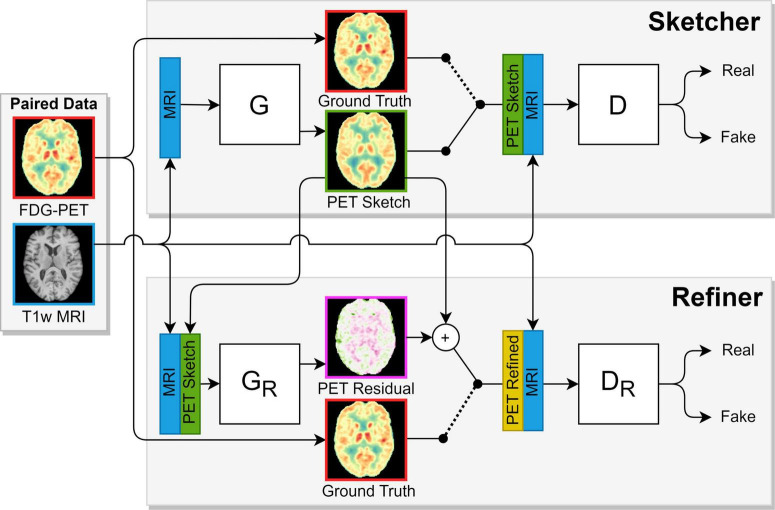
The adversarial sketcher-refiner framework inspired by Gousias et al. (2008), Jenkinson et al. (2012). The sketcher generator (G) predicts a [^18^F]FDG 3D patch (PET Sketch) from an MR 3D patch, and the refiner generator (G_R_) enhances this to a final synthetic image patch (PET Refined). During training, the sketcher discriminator (D) is shown patches from MR, PET Sketch, and real PET (Ground truth) images. Likewise, the refiner discriminator is shown patches from MR, PET Refined, and real PET images. The discriminators try to distinguish real from synthetic images, which motivates the generators to synthesize compelling PET patches.

The deep convolutional model was inspired by Kläser et al. (2018) and Wei et al. (2018) consisted of two Pix2Pix-like conditional generative adversarial networks (cGANs); an ordinary cGAN, dubbed sketcher, used MR images to synthesize preliminary [^18^F]FDG-PET images, and an additional GAN, dubbed refiner, enhanced the said images to final predictions (Figure 2). Both cGANs shared the same 3D-unet generator-architecture and 3D Patch-Gan discriminator-architecture. Ordinary binary cross-entropy was used for the adversarial loss, and L1 for the recognition loss. In both cases, the brain-mask confined optimization to within-brain voxel predictions. Finally, the refiner generator was penalized with an additional gray matter masked L1 term to emphasize tissue relevant to neurodegenerative diseases (Garibotto et al., 2017).

To strengthen model generalization, training augmentations were applied to the PET/MRI data and associated masks in the form of random sagittal mirroring [probability (*p*) = 0.5], random affine transformations in each dimension (scale ∼ Uniform(0.9,1.1), degree rotation∼Uniform(−10, 10), *p* = 1), and random bias field artifacts (*p* = 0.5). Hyperparameters were tuned using the holdout splits, and the final sketcher-refiner model, which consisted of 200 M trainable parameters, was pre-trained on the ADNI CN cohort for 90,000 steps using ADAM and a batch size of two. The initial generator and discriminator learning rates were 10^−4^, both decayed 10-fold at step 20,000 and once again at step 50,000. Finally, the model was fine-tuned for 60,000 steps on the local CN cohort using the same initial learning rates and decay steps. All model training was performed on a single NVIDIA TITAN V graphics card.

Since the memory considerations of whole-brain networks inevitably restrict resolution or network size, our model was trained to predict 3D patches of 32 neighboring, full, sagittal slices. At inference, a total of 160 per-image overlapping patches were extracted, fed through the model, and fused to a full 192 × 192 × 192 synthetic baseline PET (sbPET). To handle overlapping slice predictions and tissue inconsistency at patch borders, a weighted average scheme was designed such that slice influence decreased with the sagittal distance to the center of the originating patch.

### Quantitative and qualitative evaluation of synthetic controls

Whole-brain synthetic accuracy was determined by comparing the CN test images with their corresponding synthetic images in terms of peak signal-to-noise ratio (PSNR), mean relative %-difference (RD%), and mean absolute-value relative %-difference (Abs%) (Wang et al., 2004). All three metrics have invariance to scale and, by extension, invariance to the PET normalization scheme. The RD% metric can be thought of as expressing bias, that is, whether the model tends to over or underestimate uptake. Similarly, Abs% can be likened to the model’s overall uncertainty, i.e., the average magnitude of deviation between synthetic and true images. RD and Abs% scores were also calculated for selected regions of the Hammersmith atlas to examine the spatial distribution of the synthetic error (Hammers et al., 2003; Gousias et al., 2008). The same metrics were plotted for the AD subjects to ratify the findings of the CN group. We then denormalized the synthetic and true images of the CN group to the original MBq/ml space and calculated the fraction of PET variance explained by each sbPET. In addition, the denormalized PET-sbPET joint uptake distribution was visualized in a histogram to reveal possible prediction biases.

### Application in the diagnosis of Alzheimer’s disease

As a proof-of-concept for a clinical application, the synthetic images were incorporated as personalized controls in a quantitative and interpretable method for locating abnormal [^18^F]FDG uptake. To ensure sbPET and PET scale consistency, each sbPET images was post-normalized by the average signal of a region of healthy uptake defined by the true PET image. More specifically, the region mask captured the gray and white matter voxels of the true PET with the top 2% uptake after a 3 mm gaussian blurring. Following this normalization, both PET and sbPET were blurred by a 3 mm gaussian, and a simple abnormality map, *Z*_*%*_, was computed as the voxel-wise relative %-difference of the true PET, *Y*, with respect to the post-normalized sbPET, $\hat{Y}$. That is, the *Z*_*%*_ value at some voxel coordinate, *x*, is computed as:


$$Z_{\%}\,\left(x\right)=\frac{Y\,\left(x\right)-\hat{Y}\,\left(x\right)}{\hat{Y}\,\left(x\right)}\cdot 100\%$$

This quantitative abnormality map can be interpreted as the deviation of the subject’s [^18^F]FDG uptake from the personalized baseline. The essential strength is that the model is trained to synthesize sbPET images of healthy appearance taking brain anatomy into account. So for a healthy subject, the sbPET and PET should be similar even in the presence of atrophy or irregular morphology. In contrast, for a patient with AD, the map should express abnormality in areas of metabolic dysfunction, as the anatomical context by itself would not explain the drop in true uptake.

To investigate clinical applicability, abnormality maps were calculated for the CN and AD test subjects. The maps were nonlinearly registered to MNI space and averaged within groups to highlight differences between CN and AD subjects. Additionally, maps of selected subjects were compared with the vendor-provided statistical maps currently used in clinic. Statistical deviation maps generated by Siemens Scenium software were extracted for the true PET images using the FDG2A template (age 46–79 years, cerebellar normalization region). The two methods were qualitatively evaluated in context of diagnosing neurodegenerative disease, and special attention was put on robustness in presence of atrophy.

## Results

Model training time was split between ADNI pre-training, 48 h, and local cohort fine-tuning, 24 h. On average, a full sbPET volume took 12 s to synthesize.

### Quantitative and qualitative evaluation of synthetic controls

**FIGURE 3:**
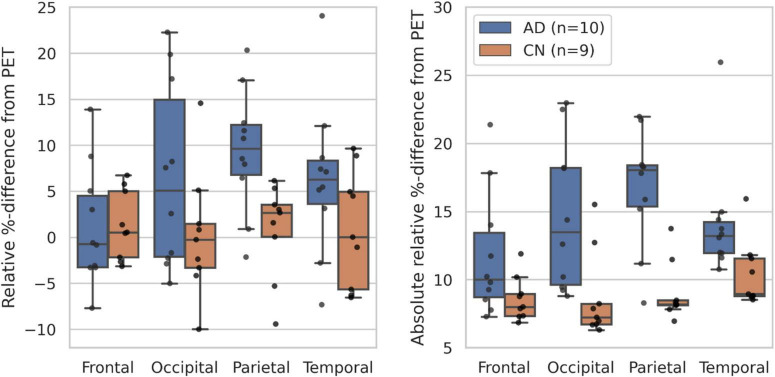
Boxplots of the RD% **(left)** and Abs% **(right)** scores within selected Hammersmith brain regions for the CN (*n* = 9) and AD (*n* = 10) test subjects (Fonov et al., 2009; Modat et al., 2010).

**FIGURE 4:**
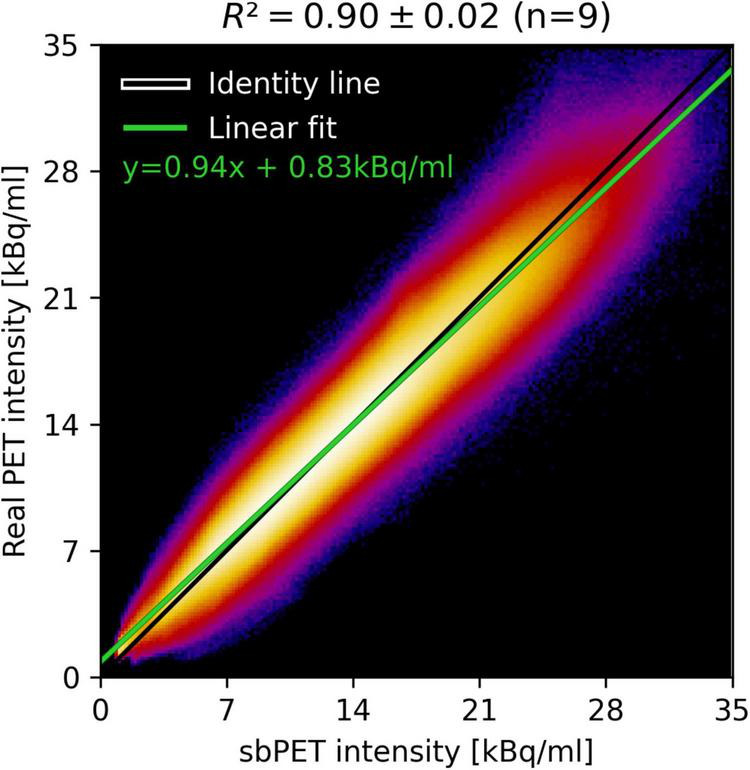
Summed joint histogram of predicted and true uptake for within-brain voxels of the CN test group and the corresponding linear fit (green). The average explained variance (*R*^2^) is calculated for the linear fit with the standard deviation as uncertainty measure.

The fine-tuned synthetic model obtained an average score of 26.3 ± 1.0 PSNR, 0.5 ± 4.7% RD%, and 9.4 ± 1.9% Abs% on the nine CN test subjects, which was an improvement over the pre-trained model across all metrics (Supplementary Table 4). The small magnitude RD% suggests an unbiased prediction of uptake in cognitive normal subjects, and the corresponding Abs% score establishes an average voxel error magnitude of less than 10%. Figure 3 depicts group-average RD and Abs% within Hammersmith atlas brain regions for both the CN and AD group (Hammers et al., 2003; Gousias et al., 2008). Evidently, the RD% error is spatially uniform and zero-centered across regions for the CN cohort, but consistently large (positive) for the AD cohort. This observation of overestimation of uptake in subjects with neurodegenerative disease is expected, as the synthetic images reflect the high metabolism associated with *healthy* brain activity. The deviation is especially notable in the parietal lobe, which is a region often associated with dementia pathology (Brown et al., 2014). For the region-wise Abs% scores, the CN group exhibits an average error of 7–16%, while the error of the AD group is significantly larger (8–26%) and dependent on brain region. The joint histogram of [^18^F]FDG uptake in Figure 4 shows a high correlation between predicted and true activity in CN subjects (*R*^2^ = 0.90 ± 0.02), shows no systemic effect in the residual, and the linear fit is close to the theoretically optimal identity line.

### Application in the diagnosis of Alzheimer’s disease

**FIGURE 5:**
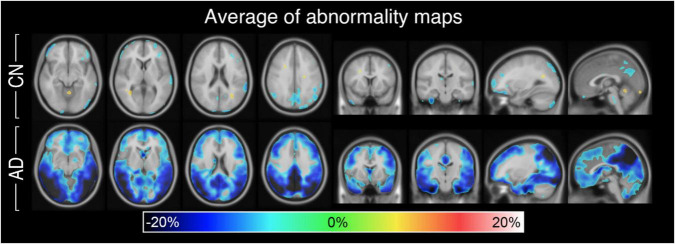
Abnormality maps of the CN group (*n* = 9) and AD group (*n* = 10) nonlinearly registered to MNI space and averaged. On average, the personalized method finds little to no abnormality for the CN group but significant hypometabolism in the gray matter of the AD group.

Abnormality maps were generated for the 9 CN and 10 AD subjects, nonlinearly registered to MNI space, and averaged within group (Figure 5). The abnormality metric is found to be spatially uniform for CN subjects with an average close to zero (no abnormality) almost everywhere. Contrarily, the AD group exhibits significant hypometabolism (15–20% reduced uptake on average) in the parietotemporal and frontal region indicative of neurodegenerative disease.

**FIGURE 6:**
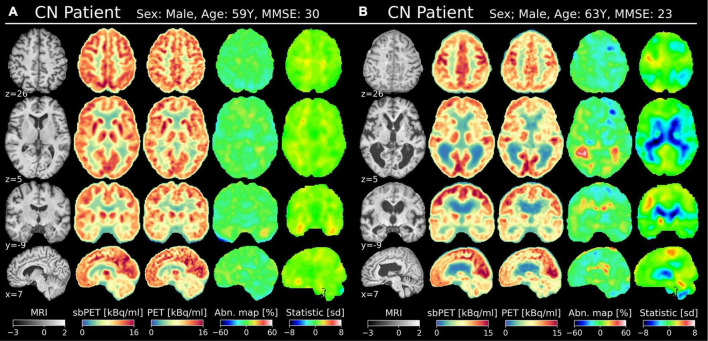
Two CN subjects in MNI space (affine transformation); one with minimal atrophy **(A)** and one with substantial atrophy **(B)**. The MNI coordinate is denoted for each view. Columns from the left: T1w MRI (normalized intensity), sbPET (post-normalized), true PET, abnormality map, vendor-provided statistical map.

**FIGURE 7:**
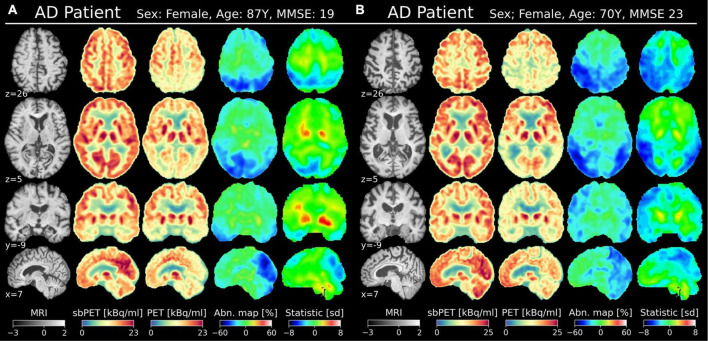
Two AD subjects in MNI space (affine transformation); one with minimal atrophy **(A)** and one with substantial atrophy **(B)**. The MNI coordinate is denoted for each view. Columns from the left: T1w MRI (normalized intensity), sbPET (post-normalized), true PET, abnormality map, vendor-provided statistical map.

Figures 6, 7 present PET, sbPET, abnormality maps, and vendor-provided statistic maps for four test subjects: Two CN subject; one with and without atrophy (Figure 6) and two AD subjects (Figure 7). Note that in the less challenging case of healthy subject A without atrophy, both the abnormality map and the vendor-provided map correctly predict healthy (green) whole-brain uptake (Figure 6A). In presence of atrophy, however, the template-based method incorrectly determines abnormality in areas of ventricular enlargement (Figure 6B). With a patient-specific design, the abnormality map avoids this issue altogether, as the conditioning on anatomy allows the synthetic images to account for reduced uptake in areas of atrophy. In both subjects with Alzheimer’s disease, our method confidently localizes areas of abnormal uptake (Figure 7). Note the estimated 40–60% reduction in uptake in the parietotemporal gray matter region, and how the method correctly predicts reduced metabolism in wide fissures (Figure 7).

## Discussion

This study demonstrated the feasibility of a novel deep learning method for synthesizing healthy [^18^F]FDG-PET images from patients’ own MR images. Through transfer-learning, the model conformed to the high-resolution images of modern PET/MRI systems, making the synthetic images well suited for clinical tasks. As a proof-of-concept, one such clinical application was showcased by employing the synthetic images as personalized, healthy baselines in the diagnosis of AD. Compared with vendor-provided statistical maps, the personalized abnormality maps appeared more robust to heterogeneous brain morphology. Consequently, this methodology may improve differential diagnosis in elderly populations with atrophy.

The synthetic images accurately reflected healthy [^18^F]FDG-PET appearance and proved to be resistant to anatomical variance. Figure 3 (left) shows a small, spatially uniform synthetic error of ±10% for CN subjects. Likewise, Figure 3 (right), Figure 4 suggest that the model prediction is unbiased with respect to brain region and true uptake. It is difficult to compare the results directly with related studies due to the small test set and intrinsic differences in datasets, validation schemes, synthetic resolution, and use of scale variant metrics, however, a PSNR of 26.3 is considered high given the target resolution (Manjooran et al., 2021). Combined, these observations suggest that the model is both accurate and robust in its prediction of healthy uptake, which is necessary for the images to act as personalized baselines. The key finding is embodied by CN test subject B, for whom the uptake prediction remains accurate despite presence of both cortical and ventricular atrophy (Figure 6B). Finally, the performance gap gained through transfer-learning stresses the importance of including high-resolution scans in the training cohort (Supplementary Table 4).

Synthetic controls may be more suitable for clinical applications compared to conventional computational methods. In the context of machine learning assisted diagnosis, a popular approach has been to classify the disease directly from the imaging data (Arbabshirani et al., 2017; Wen et al., 2020). For several reasons, this classification methodology may be unfit for clinical use; the first being that prediction reasoning is inherently difficult to interpret from neural networks. Secondly, such models may be limited by the cohort on which they are trained as comorbidities and rare disorders are often not be represented in the dataset. Finally, binary classifiers frequently ignore the underlying heterogeneity of each disorder, that is, differences in the subtype and magnitude of the disease (Arbabshirani et al., 2017). Usually, the optimal treatment path varies according to these disease specifications, so the limited information provided by classification-based models may be less actionable in clinic (Arbabshirani et al., 2017). The methodology embodied by synthetic controls largely avoids these issues. Class-imbalance is circumvented as the model only needs to characterize a single population: Healthy individuals. Additionally, the simple interpretation and versatility of a synthetic control permits it to complement existing clinical routines rather than replacing them. Finally, instead of having to train individual models for each clinical task, the same sbPET can act as a high-dimensional feature in multiple neuroimaging applications.

As a proof-of-concept for one such application, synthetic images were incorporated into a personalized abnormality map to aid the diagnosis of neurodegenerative disorders. The current diagnostic methods of AD are not ideal as vendor-provided statistical tools can interpret healthy, age-related atrophy as abnormal uptake (Brown et al., 2014). Such is the case for CN subject B of Figure 7 for whom the vendor-provided statistical map incorrectly predicted hypometabolism in areas of ventricular and cortical atrophy. The personalized map showed less pronounced abnormality as the healthy synthetic control accounted for the localized drops in uptake caused by atrophy. This finding is further confirmed by the abnormality map group-average, which is spatially uniform and zero-centered for the CN test group (Figure 5). Importantly, the personalized map remains sensitive to AD pathology, as AD maps, on average, show abnormal metabolism in the parietotemporal and frontal gray matter regions. For such subjects, one may more confidently assume that illness is the cause of reduced uptake, as the healthy sbPET images are strikingly different from the true PET images. Although the results are based on a small test set, this separation of atrophy and disease demonstrates how a personalized control may enhance the diagnosis of AD.

The methodology and advantage of personalized diagnostic models is not a novel discovery. Burgos et al. (2017) and Choi et al. (2019) propose pipelines for diagnosing neurodegenerative disorders based on similar personalized, healthy controls. Burgos et al. (2017) designed a semi-personalized model, which comprised scans of subjects anatomically similar to the patient. An abnormality map was then computed by comparing the model and ground truth through a quantifiable *Z*-score. Choi et al. (2019) suggested a fully personalized method by having a VAE synthesize the [^18^F]FDG-PET control in an unsupervised manner and subsequently detect abnormality *via* a mean square error metric. Our study builds on these strengths by proposing a novel method that is fully personalized, quantifiable, and optimized on a clinical dataset. Importantly, the interpretable abnormality map differentiates between hyper- and hypometabolism and the synthetic process is void of any cohort data and nonlinear registrations tools, which are often vulnerable to atrophy.

The sbPET image may be deployed as a general tool in other neuroimaging tasks and standard clinical routines. Rajagopal et al. (2021) proposed the use of a deep-domain translated image as a prior in the reconstruction of PET images from sinograms. More specifically, a synthetic [^18^F]FDG-PET could impose sparsity constraints on the reconstruction problem to allow recovery of noisy low-dose PET/MRI. For the alignment of multimodal images, a synthetic image could transform a challenging PET-to-MRI registration to a unimodal PET-to-synthetic-PET-registration problem (Han, 2017; Fu et al., 2020). Lastly, the abnormality map approach may be extended to the diagnosis of other disorders, for instance, in the segmentation of brain tumors and localization of epileptic foci. However, further work is required to explore these implementations (Sarikaya, 2015).

This study posed a number of limitations. Although the ADNI-cohort is heterogenous, all scans of the local cohort were performed at one hospital with the same PET/MRI system, which could reduce generalizability to different scanners. Notably, the test images were acquired simultaneously by a hybrid PET/MRI system and were thus geometrically aligned, which may not be the case for sequentially acquired images. Furthermore, the CN subjects of the local cohort were examined due to some suspicion of disease, perhaps arisen from a behavioral or cognitive change (Weller and Budson, 2018). Although cognitive impairment was ruled out by clinical experts, it could be argued that the images of the CN subjects do not resemble the uptake of healthy individuals. One example is CN subject B whose MMSE score alone would suggest mild cognitive impairment (Figure 6B; Weller and Budson, 2018). The presence of epilepsy, vascular infarcts, and other conditions in the fine-tuning training data did, however, not hinder the abnormality map sensitivity toward AD uptake patterns (Figure 5). Nevertheless, to ensure model generalizability in a clinical setting, stricter inclusion criteria like that of ADNI should be imposed, and the clinical dataset should be extended to encompass multiple scanners and a wider demographic. Importantly, a significantly larger test set is required to validate the proof-of-concept application in AD.

The pre-processing pipeline was a limitation as the tools for skull-stripping, intra-patient registration, and gray matter segmentation were challenged by severe atrophy. Data quality often dictates model performance, so imperfections in the pre-processing stage may lead to inadvertent model penalization and a reduction in synthetic accuracy (Han, 2017). Particularly, the normalization method was less consistent in subjects with extensive ventricular systems, since the large proportion of CSF resulted in a too low normalization constant. Although post-normalization of the synthetic images mitigated most of the intensity issues in relation to the abnormality map, the effect of alternative normalization methods should be investigated. In future studies, skull-stripping could possibly be omitted altogether by implementing a minimal pre-processing approach. Image misalignment may be improved by replacing the multimodal MRI-to-PET registration with a simpler unimodal PET-to-sbPET registration as suggested by Wen et al. (2020).

## Conclusion

The aim of this work was to develop and test a model that synthesizes individual healthy [^18^F]FDG-PET images to be used as patients’ own controls in neuroimaging tasks. This was achieved through a synthetic model that deployed a 3D convolutional neural network to predict [^18^F]FDG-PET images from patients’ own MR images. The results showed that the proposed deep learning model reliably produced healthy-appearing images for cognitive normal subjects. As a proof-of-concept for a clinical application, the model was deployed in a personalized abnormality map for diagnosing AD. Ultimately, this approach may strengthen patient examination by eliminating dependence on statistical databases that currently impede the diagnostic process. Before the model might be utilized in clinic, further investigations should be made in model generalization and task-specific implementation of the synthetic controls.

## Data availability statement

The datasets presented in this article are not readily available because of restrictions on sharing patient data. The ADNI data is available online upon request. Requests to access the datasets should be directed to adni.loni.usc.edu.

## Ethics statement

Use of patient data was approved by the Danish Patient Safety Authority (ref. 3-3013-1513/1). Written informed consent for participation was not required for this study in accordance with the national legislation and the institutional requirements.

## Author contributions

CH, CL, and FA were the primary contributors to the conception and design of the study. UL, IL, and OH contributed to the conception and design of the study. OH and IL analyzed the patient scans. CH wrote the initial draft, which was revised by CL and FA. All authors contributed to the final manuscript revision, read, and approved the submitted version.

## Footnotes

1
adni.loni.usc.edu

2 www.adni-info.org for criteria.
