# Supplementary material for: A zero-dose synthetic baseline for the personalized analysis of [18F]FDG-PET: Application in Alzheimer’s disease
Source: Front Neurosci. 2022 Nov 24;16:1053783. doi: 10.3389/fnins.2022.1053783 (PMC9749397; doi:10.3389/fnins.2022.1053783)
Supplement: Supplementary file 1 [file Data_Sheet_1.docx]

# **SUPPLEMENTARY MATERIAL**

| **Study count**  **(122 total)** | **Repetition**  **time [ms]** | **Echo time [ms]** | **Flip angle [degrees]** | **Voxel size [mm³]** | **Matrix Size** |
| --- | --- | --- | --- | --- | --- |
| 117 | 1900 | 2.44 | 9 | 1.0 x 0.49 x 0.49 | 192 x 512 x 512 |
| 2 | 2200 | 2.91 | 10 | 1.1 x 0.55 x 0.55 | 208 x 512 x 512 |
| 2 | 1900 | 2.43 | 9 | 1.0 x 0.51 x 0.51 | 192 x 512 x 512 |
| 1 | 1900 | 2.46 | 9 | 1.0 x 0.47 x 0.47 | 192 x 512 x 512 |

**Table 2** MRI acquisition parameters for the 122 T1w MPRAGE scans in the local cohort. The scans were initially acquired with voxel size 1.0 x 1.0 x 1.0 mm^3^ and subsequently interpolated to the listed voxel size.

| **Software/Package** | **Version** | **Role** |
| --- | --- | --- |
| Scenium VB | VB60A | Vendor software - used for FDG-PET quantification. |
| NiftyReg | 1.5.58 | Registration |
| HD-BET | 1.0 | Segmentation |
| ANTs | 2.3.5 | Registration |
| FSL | 6.0.1 | Segmentation |
| Python | 3.8.10 |  |
| torch (PyTorch) | 1.10.0+cu113 | Deep learning framework |
| pytorch_lightning | 1.7.7 | High-level interface for PyTorch |
| torchio | 0.18.46 | Data I/O and medical image augmentations |
| nipype | 1.8.5 | Interface to neuroimaging software and parallelized preprocessing |
| numpy | 1.23.3 | Statistical analysis |
| hydra-core | 1.2.0 | Reproducibility |
| matplotlib | 3.6.0 | Figures |
| wandb | 0.13.4 | Logging and monitoring |

**Table 3** The main software and Python packages used, their version, and their specific role in the development and testing of our method.

| **Model** | **Cohort** | **Split** | **Studies** | **↓RD%** | **↓Abs%** | **↑PSNR** |
| --- | --- | --- | --- | --- | --- | --- |
| Pretrained | ADNI CN | *Train* | 512 | 1.0 ± 5.2 | 10.2 ± 2.7 | 25.3 ± 1.4 |
|  |  | *Validation* | 88 | 0.3 ± 4.7 | 9.7 ± 2.2 | 25.2 ± 1.3 |
|  | Local CN | *Train* | 75 | 3.9 ± 4.0 | 12.3 ± 1.7 | 23.6 ± 0.8 |
|  |  | *Validation* | 19 | 3.5 ± 6.5 | 12.6 ± 3.9 | 23.8 ± 1.0 |
|  | Local CN | *Train* | 75 | 1.3 ± 3.3 | 8.3 ± 1.6 | 27.2 ± 1.1 |
| Finetuned |  | *Validation* | 19 | 2.0 ± 3.2 | 9.3 ± 1.9 | 26.0 ± 1.3 |
|  |  | *Test* | 9 | 0.5 ± 4.7 | 9.4 ± 1.9 | 26.3 ± 1.0 |

**Table 4** Performance metrics at inference of the pretrained and finetuned models on the different data cohorts and splits. Standard deviation is used as uncertainty measure.
